# Clinical Application of Circulating Tumour Cells in Prostate Cancer: From Bench to Bedside and Back

**DOI:** 10.3390/ijms17091580

**Published:** 2016-09-20

**Authors:** Luis León-Mateos, María Vieito, Urbano Anido, Rafael López López, Laura Muinelo Romay

**Affiliations:** 1 Axencia Galega de Coñecemento en Saúde (ACIS), SERGAS, Avda, Fernando de Casa Novoa, Santiago de Compostela 15707, Spain; 2 London Regional Cancer Program, London Health Sciences Centre, London, ON N6A 4L6, Canada; mariavieito@gmail.com; 3 Translational Medical Oncology/Liquid Biopsy Analysis Unit, Health Research Institute of Santiago (IDIS), Complexo Hospitalario Universitario de Santiago de Compostela (SERGAS), Trav. Choupana s/n, Santiago de Compostela 15706, Spain; urbanoanido@gmail.com (U.A.); rafa.lopez.lopez@gmail.com (R.L.L.)

**Keywords:** prostate cancer, circulating tumour cells, tumour markers, precision oncology

## Abstract

Prostate cancer is the most common cancer in men worldwide. To improve future drug development and patient management, surrogate biomarkers associated with relevant outcomes are required. Circulating tumour cells (CTCs) are tumour cells that can enter the circulatory system, and are principally responsible for the development of metastasis at distant sites. In recent years, interest in detecting CTCs as a surrogate biomarker has ghiiukjrown. Clinical studies have revealed that high levels of CTCs in the blood correlate with disease progression in patients with prostate cancer; however, their predictive value for monitoring therapeutic response is less clear. Despite the important progress in CTC clinical development, there are critical requirements for the implementation of their analysis as a routine oncology tool. The goal of the present review is to provide an update on the advances in the clinical validation of CTCs as a surrogate biomarker and to discuss the principal obstacles and main challenges to their inclusion in clinical practice.

## 1. Introduction

Prostate cancer (PCa) is the most common solid tumour and represents 10% of all male cancer deaths. This tumour exhibits important variability in its evolution, ranging from indolence to rapid growth, dissemination, and lethality. Current treatment options for localised PCa include active surveillance, surgery (radical prostatectomy), external beam radiation therapy and interstitial radiation therapy (brachytherapy) [1]. Typically, patients respond to androgen ablation; however, many eventually become castration-resistant and develop skeletal metastasis. While localised disease can be treated effectively, there are no truly curative therapies for castration-resistant disease.

Currently, histopathological analysis (Gleason score) and serum prostate-specific antigen (PSA) levels are the key determinants of therapeutic decision-making. However, PSA has some weaknesses as a biomarker, as it is also increased in benign prostatic hyperplasia; PSA levels can be similar in indolent and aggressive cancers and often do not accurately indicate patient response to a given treatment. In addition, histopathological analysis is inadequate for predicting disease evolution [2]. Therefore, the clinical application of new surrogate markers will provide the opportunity for improving patient management and therapeutic selection and monitoring.

For clinical use, a novel biomarker should provide relevant information to clinicians cost-effectively. Therefore, the standards or at least the accuracy of new biomarkers should be improved. The ideal biomarker should be determined using accessible biopsy specimens such as peripheral blood or urine, and provide evidence of the patient outcomes (prognosis marker) or predict the likelihood of response/benefit to a specific therapy (predictive marker) [3]. During haematogenous spread in PCa, tumour cells travel through the blood vessels, and after extravasation, colonise distant target organs (typically the bone). As circulating tumour cells (CTCs) are an intermediate population between the primary tumour and metastasis, they are candidate surrogate markers that can be measured in blood [4]. CTC quantification has experienced rapid technological development in the past few years, enabling the accumulation of important data to establish the potential clinical value of CTCs as early detection, diagnostic, prognostic, predictive, surrogate, stratification and pharmacodynamic biomarkers in different carcinomas [5,6,7]. Currently, CTCs are widely recognised as a prognostic biomarker of PCa. Numerous studies have demonstrated the association between baseline CTC levels and clinical outcomes in patients with metastasis [8,9,10,11]. In addition, decreased CTC numbers after therapy are associated with longer overall survival (OS), similar to the benefit correlated to a substantial PSA decrease or radiographic response [9,12,13]. Furthermore, changes in CTC levels usually precede PSA fluctuation, indicating even greater value in monitoring CTC numbers when changes in PSA levels or metastasic bone disease are difficult to evaluate [10]. Despite the promising data regarding the value of CTCs as a disease marker in PCa, their clinical utility should be carefully interpreted. Here, we summarise the recent advances in CTC analyses together with their clinical development, highlighting the relevance of providing comprehensive CTC molecular characterisation to achieve precision oncology in PCa, and focusing in particular on obstacles to their use in the clinic.

## 2. Current and New Approaches for CTC Detection: Application in PCa

Given the difficulty in acquiring biopsy specimens from patients with only bone metastasis and the value of identifying new biomarkers (which improve PSA information) that could be used as surrogates for survival in clinical trials, PCa presents the ideal scenario for CTC research and clinical development. Chronologically, the initial approaches in CTC detection in patients with PCa were based on reverse transcription-PCR (RT-PCR) for detecting prostate-specific [14,15] or epithelial-specific markers in non–pre-enriched blood samples. These studies demonstrated the possibility of distinguishing patients from controls based on mRNA levels of the markers, but often could not show a direct prognostic relationship. The potential additional limitations of this approach are low sensitivity due to white blood cell contamination, and variability in the copy number of a given mRNA between cancer cells.

The critical factor for CTC studies is that they are present at very low frequency in the bloodstream; it is generally estimated that there is 1 CTC per million leukocytes [16]. Given the low concentration of CTCs in the blood, extremely sensitive and specific strategies are required to process blood samples in a short period. CTC enrichment methods are mainly based on physical or biological cell properties such as size or specific marker expression (Table 1).

Immunocytochemical enrichment is the most frequently employed strategy for isolating CTCs from blood cells. These affinity techniques typically use antibodies that recognise antigens expressed by CTCs but not by blood cells. The antigen used most often for isolating CTCs is EpCAM (epithelial cell adhesion molecule), an epithelial marker overexpressed in some carcinomas [17,18]. However, it is well known that the epithelial signature of CTCs is altered during metastasis, interfering with the use of EpCAM as a universal marker [19,20,21]. Therefore, much effort has been focused on characterising and identifying additional markers to distinguish CTCs from their counterparts in blood and that reflect the phenotype heterogeneity of these cells. For example, CellSearch has US Food and Drug Administration (FDA) clearance for CTC quantification in patients with metastasic prostate cancer (mPCA) or MagSweeper only isolate EpCAM-positive CTCs [22], while other technologies, such as AdnaTest, already include epithelial–mesenchymal transition (EMT) markers to isolate CTCs from patients with PCa [23].

An important point in CTC immunoisolation is that PCa cells display tissue-specific antigens, such as PSA and PSMA, usually absent in non-epithelial cells, together with epithelial markers such as EpCAM [8]. In addition, in CTCs from PCa, EpCAM expression levels are higher than that from CTCs from other tumours, even assuming that the CTC population is heterogeneous, and some of these cells could show EMT-like characteristics [20,38].

Also in the group of antigen-dependent strategies for enriching CTC is the negative depletion of blood cells using protocols such as RosetteSep (StemCell Technologies, Vancouver, BC, Canada) [34] and in vivo isolation using CellCollector (GILUPI, Postdam, Germany), a medical wire coated with EpCAM antibodies that is inserted for 30 min in to a peripheral vein that demonstrates high CTC detection efficiency in breast and lung cancers [31,39].

Other methods for enriching or isolating CTCs are based on size, density, electrical charge or deformability. Several platforms using size as the isolation method for detecting CTCs from the blood have been reported in recent years [32,40,41]. An example of these commercially available devices is ISET (RareCells, Paris, France) or Metacell (Ostrava, Czech Republic) [26,27,40,42], filters that retain larger CTCs while allowing smaller leukocytes to pass through pores of varying geometries and size. The disadvantage of these systems is that CTC purity is low, in most cases requiring further enrichment, and the fact that leucocytes can overlap in size with CTCs. An alternative, highly used approach, even as a pre-enrichment method, is based on generating density gradients by centrifugation using Ficoll [43,44]. The principle is to enable marker-independent cell selection but it leads to high loss of tumour cells, yielding false negative results in clinical samples.

On the other hand, microfluidics have been demonstrated to be valuable platforms for CTC analyses that can be integrated into other processing steps to fully automate sample processing. These systems combine both physical and biological strategies. Microfluidic devices based on affinity selection typically yield higher purities compared with size-based selection but at the expense of throughput [18,45,46]. For example, the CTC-iChip isolates viable CTCs based on affinity to anti-EpCAM-coated microspots under controlled laminar flow conditions. This approach demonstrates higher sensitivity, selectivity and yield compared with techniques based only on immunomagnetic beads in PCa [18,37]. Another microfluidic device used in PCa is the NanoVelcro CTC Chip, composed of a silicon nanowire substrate (SiNW) coated with anti-EpCAM antibodies and an overlaid polydimethylsiloxane (PDMS) chaotic mixer that allows CTC capture. This system has shown value for enumerating and monitoring CTCs to follow responses to different treatments and to predict disease progression in PCa [35,47].

Of these technologies, CellSearch is the only technology approved by the US FDA for quantifying CTCs in metastatic breast, colon and prostate cancers [5,10,48]. Although many new technologies have been presented for CTC detection and molecular profiling in recent years, the majority have yet to demonstrate clinical utility. Currently, there is great interest in developing microdevices that can process smaller volumes of blood, decreasing assay time and cost. Conversely, some technologies are now directed at analysing larger blood volumes, particularly in early-stage tumours where the presence of CTCs is even more infrequent [31]. Overall, efforts are directed towards developing devices for live cancer cell detection with single-cell sensitivity, high selectivity and reproducibility, easy fabrication and low cost. Stringent clinical validation of new devices is now required before their introduction to the management of patients with cancer.

## 3. Realities and Challenges for CTC Molecular and Functional Characterisation in PCa

### 3.1. Molecular Profiling

CTC level is a strong predictor of OS and has predictive value for patients with metastatic castration-resistant PCa (mCRPC). Nevertheless, the majority of trials have focused on the clinical utility of CTC enumeration, using platforms that detect CTCs expressing epithelial markers. As mentioned earlier, this approach is simplistic, as it excludes tumour stem cells, CTC clusters and CTCs with mesenchymal or anaplastic phenotypes, which may have important prognostic and predictive implications [49]. Previous works have demonstrated the feasibility of transcriptional and genomic profiling in CellSearch-detected CTCs from patients with PCa [17,50]. Androgen receptor (AR) chromosomal amplifications have been detected in CTCs from patients with mCRPC by fluorescent in situ hybridisation (FISH). Importantly, the impact of *TMPRSS2*-*ERG* rearrangements has also been studied using CTC. FISH detection of *ERG* rearrangements was significantly associated with the magnitude of PSA decline in chemotherapy-naïve patients treated with abiraterone [51]. In another study, Dittamore et al. analysed 48 samples from 21 patients with mCRPC treated with abiraterone plus prednisone (43%) or enzalutamide (57%) [52]. In that study, no responses were seen in patients with high AR expression on CTCs, while 53% of patients with low AR had decreased PSA and stable radiographic disease.

CTC molecular and genomic profiling may identify novel mutations, shed light on mechanisms of resistance to therapy and aid in predicting the likelihood of response to a given therapy in real time and for specific patients. In this context, AR splice variant 7 (AR-V7) expression in EpCAM-positive CTCs from men with progressive mCRPC was recently associated with resistance to abiraterone and enzalutamide, while no correlation was found between the presence of ARV7 mRNA in CTCs and the response to taxanes [53,54,55]. These studies demonstrate that AR-V7 expression in CTCs represents a valuable tool for guiding treatment selection in mCRPC.

Importantly, CTCs are entirely different from almost all other biomarkers because they represent a sampling of a patient’s tumour, and can subsequently reflect the heterogeneity of metastatic sites. For example, PSMA expression patterns between primary tumour and CTCs differ [39]. The option of developing single-cell analyses of CTCs allowed in-depth analysis of tumour heterogeneity in mPC. Using this strategy Miyamoto and collaborators analysed primary tumours, CTCs and metastasis using RNA sequencing (RNA-seq) and found considerable heterogeneity, including the expression of *AR* gene mutations and splicing variants. In addition, in patients progressing to AR inhibitors, Miyamoto and colleagues observed the activation of non-canonical Wnt signalling that could contribute to treatment failure [56]. Using whole genome sequencing on single CTCs obtained with the NanoVelcro CTC Chip with laser capture microdissection (LCM), Jiang and collaborators found that 86% of the clonal mutations identified in CTCs could be traced back to either the primary or metastatic tumour. However, they also determined highly heterogeneous short structural variants in *PTEN*, *RB1* and *BRCA2* in all tumour and CTC samples [57].

These and other results lead us to believe that not only CTC count but also their molecular characterisation may be of value for response monitoring and drug selection in patients with mPCa.

### 3.2. CTC in Vitro/in Vivo Expansion

One of the main challenges in the field of CTC development is the possibility of expanding the cells in vitro*/*in vivo. This would permit better characterisation of CTCs from patients to disease progression, for example in individualised anti-tumour therapies. Although achieving CTC culture in vitro remains difficult, some promising results have emerged [58] and support the idea of a new era in oncology. For example, one prostate CTC line was established from more than a hundred CTCs following the depletion of blood components using cell aggregation, followed by culture in vitro for more than 9 months under 3D conditions. The CTC line was tumorigenic in mice, with a similar mutation pattern to that present in lymph node metastases in the patient from which it was derived [59]. In addition, PCa cells are recoverable in murine models using CTC-iChip and can then be cultured in vitro [60].

A complementary strategy for expanding CTCs is through the use of mouse models. Patient-derived xenograft (PDX) models are being used in cancer research. Williams and collaborators isolated CTCs from mPCa patients using density gradient centrifugation followed by red blood cell lysis and flow cytometry depletion of CD45-positive mononuclear cells. CTCs were then injected into immunocompromised mice, generating mouse xenografts [61]. Positive results were also obtained with CTCs isolated using CellSearch. CTCs were isolated from six patients with mPCa and the xenograft assay was developed in 8-week-old nonobese diabetic/severe combined immunodeficiency (NOD/SCID) mice that were subcutaneously injected with increasing amounts of CTCs (50–3000). Human CTCs were found in eight of eight murine peripheral blood samples and in six of eight murine bone marrow samples after a median follow-up of 10.3 months. This study showed a higher rate of xenograft generation success than that previously reported in breast cancer and hepatocellular carcinoma and evidence that low quantities of EpCAM-positive CTCs putatively contain metastasis-initiating cells (MICs) [62].

Despite these promising results, the expansion of CTCs remains a major challenge in PCa and other tumours. In fact, there is a dire need for standardised protocols to resolve the limitations of CTC culture, such as the low numbers of CTCs isolated and their viability after enrichment.

## 4. Clinical Development of CTCs as a Biomarker of PCa

### 4.1. Requirements for Biomarker Validation

The first effort to standardise the requirements for the clinical validation of biomarkers was the development of common guidelines for reporting the results of studies on tumour markers [63], i.e., the REMARK consensus (REporting recommendations for tumour MARKer prognostic studies) following the recommendations of the NCI-EORTC First International Meeting on Cancer Diagnostics in 2001.

In 2004, the FDA Critical Path Initiative identified the lack of adequate biomarkers as an obstacle to drug development and promoted the foundation of a ACCR-FDA-NCI-backed Cancer Biomarkers Collaborative (CBC), a consortium founded to ‘accelerate the translation of cancer therapeutics into the clinic by shaping the processes for the effective development of validated biomarkers and their use in clinical trials’. In 2010, the CBC committed to 27 recommendations for the clinical validation of new molecular biomarkers [64]. These recommendations focus on eight different areas for improving biomarker development: biospecimens, analytic performance, standardisation and harmonisation, bioinformatics, collaboration and data sharing, stakeholder education and communication, regulatory issues and science policy.

Clearer than the CBC recommendations is the proposal of the Evaluation of Genomic Applications in Practice and Prevention (EGAPP) working group, which consists of three successive requirements: analytic validity, clinical validity and clinical utility [65]. While analytical validity refers to the reproducibility, accuracy and reliability of a test, clinical validity necessitates that the test can identify the phenotype, disease or subgroup of patients of interest. Therefore, clinical validity encompasses analytical validity but also has to take into account the specificity and the disease prevalence to calculate the positive and negative predictive value. Lastly, clinical utility requires that the use of a test provide the added benefit of patient management and treatment decision-making.

Although the level of evidence for each of these components can be evaluated objectively based on pre-specified benchmarks [66], it is important to note that clinical utility requires both clinical and analytical validity. In this sense, a test validated in a randomised controlled trial also has to perform reliably in external quality assessments in order to be useful for clinical use.

Finally, a recent document released by the European Group on Tumor Markers established a four-phase model for biomarker validation analogous to the pathway for therapeutic trials in oncology [4]. This pathway clarifies the strategies and objectives in each step of the process and provides several recommendations to guide trial design for validating a tumour biomarker.

### 4.2. Obstacles to the Clinical Use of CTCs

#### 4.2.1. Analytical Validation

From an analytical point of view, a validated method has to show high reproducibility of results in different measures from the same patient, but this is not always possible for CTC enumeration.

One of the main sources of variation is the low concentration of CTCs in blood, where up to 30%–40% of patients with metastasis and more than 90% of patients with localised disease do not have >5 CTCs per 7.5 mL blood [67]. Thus, a difference of one CTC between different measures could involve the classification of patients in the group for good vs. poor prognosis. A potential means of improving CTC yields even without improving sensitivity would be using larger blood samples, increasing the number of expected CTCs [68].

The sensitivity of a given method is typically tested using tumour cell lines mixed with blood. Patient-derived CTCs are smaller, more heterogeneous and may express different markers from cancer cells in culture [69], and a better surrogate, such as cells from primary tumours, should probably be employed. In this sense, the absence of epithelial markers in CTC samples due to EMT impairs the recovery rate in patients with potential poorer prognosis.

Another critical source of error is the variability in the analytical method chosen. In this regard, CellSearch data from 14 different laboratories showed that although the assay had high inter-instrument and inter-assay reproducibility, inter-laboratory reproducibility was low due to inter-observer variation [70]. Other studies have reproduced this finding [71].

Recent studies comparing different methods in paired samples from the same patient have shown that microfluidic systems and other methods that are EpCAM expression-independent can isolate a higher number of CTCs compared with CellSearch, even in samples that are negative for CTC counting using the reference method. Conducting additional studies focusing on quality control and benchmarking the performance and reliability of these methods is important.

#### 4.2.2. Clinical Validation

Although most methods for isolating and quantifying CTC require the prospective collection of samples, many studies demonstrate the clinical validity of convenience cohorts without a clinically significant, pre-specified primary end-point or pre-planned sample size calculation. Consequently, many studies may be underpowered (1-B < 0.8) for verifying a clinically relevant prognostic effect or are confused by too-permissive inclusion criteria [72].

Another potential issue is the fact that although CTC isolation techniques are highly specific (>98% for most techniques), their sensitivity remains poor. In this regard, CTC fragments have prognostic value even in patients with no conventional CTCs, suggesting the critical impact of sensitivity for the clinical validation of new technologies [73,74].

Even considering these limitations, the hazard ratio (HR) related with high CTC count represents clinical significance of sufficient magnitude, mainly in patients with PCa.

#### 4.2.3. Evidence Supporting the Clinical Use of CTCs in PCa

##### Localised Disease

Some investigators have evaluated the number of CTC in patients with localised PCa. In a small study performed in 37 patients (only eight had non-metastatic disease), the authors related a cut-off of ≥5 CTCs per 7.5 mL blood with worse survival [75]. The main limitations of the study were the small number of patients with localised disease, the wide range of PSA (from 0.2 to 22.6 ng/mL) and the absence of pathologic confirmation of staging.

Gewanter et al. detected circulating PCa cells using PSA level RT-PCR analysis in the serum of 161 patients with localised PCa treated with radiotherapy [76]. The median follow-up was 29 months. The pre-treatment RT-PCR result was not predictive of biochemical relapse-free survival or clinical disease-free survival. Pre-treatment negative RT-PCR was associated with better outcomes only in 25 patients with T3–4 PCa. In a cohort of 152 patients with localised disease, Meyer and collaborators did not find a correlation between pre-radiotherapy CTC count and PSA levels, disease characteristics or biochemical recurrence [77].

##### Advanced Disease

In 2008, de Bono and colleagues conducted the IMMC38 study in 231 patients with mCRPC treated with chemotherapy [9]. Patients with ≥5 CTCs per 7.5 mL blood were associated with worse OS (11.5 vs. 21.7 months; HR, 3.3; *p* < 0.001), which also distinguished patients into favourable and unfavourable prognosis groups. CTC counts showed even greater prognostic value than PSA levels. This study led to FDA approval of the CellSearch quantification system of CTC in advanced PCa. A follow-up study of the same cohort analysed only patients receiving first-line therapy and showed that absolute CTC count and changes in CTC count measured as continuous variables were survival prognostic factors in this group [10]. In another study, conducted in 162 patients with mCRPC who received docetaxel, CTC levels at baseline (cut-off 5 CTCs/7.5 mL blood) and at 2–5 weeks (≤5 or ≥5 CTCs/7.5 mL blood) correlated with survival, while the decline in PSA (30% or 50%) did not [78].

The randomised COU-AA-301 phase III study of abiraterone in docetaxel-refractory mCRPC confirmed the prognostic value of ≥5 CTCs per 7.5 mL blood, where chemotherapy-naïve patients with mCRPC on docetaxel and prednisone with or without lenalidomide were investigated in a randomised, double-blind, placebo-controlled phase III trial (MAINSAIL) [79]. In that study, CTCs were enumerated at baseline and during the first three cycles. CTC conversion from ≥5 CTCs per 7.5 mL blood to <5 CTCs per 7.5 mL blood along with changes in serum lactate dehydrogenase (LDH) was strongly predictive of OS. Similar results were observed in a phase III study using docetaxel with or without lenalidomide [80]. Blood samples for CTC analysis were collected from 208 patients: 105 received docetaxel plus lenalidomide (DL) and 103 received docetaxel only (D). Baseline CTC counts were <5 CTCs per 7.5 mL blood in 87 patients and ≥5 CTCs per 7.5 mL blood in 121 patients. Overall, the 2-year OS was lower in patients with baseline CTC ≥ 5 CTCs per 7.5 mL blood in both treatment arms (DL, HR 3.63, *p* = 0.0044; D, HR 3.41, *p* = 0.0459). Overall, an increase in CTCs between baseline count and cycle 4 was associated with significantly shorter OS (HR 5.24; *p* = 0.0251).

The phase III SWOG S0421 trial compared the effectiveness of docetaxel plus atrasentan versus docetaxel alone [81]. The study did not meet its co-primary end-points of improved OS and progression-free survival with the addition of atrasentan. As part of the study, CTCs were enumerated at baseline and 21 days after the first dose. Baseline CTC counts were correlated with recognised prognostic markers, including PSA, alkaline phosphatase, haemoglobin, liver disease and bone pain. The median OS was 26 months for the <5 CTCs per 7.5 mL blood group versus 13 months for patients with ≥5 CTCs per 7.5 mL blood at baseline. Unfortunately, the relationship with LDH was not assessed.

Beyond the value of single baseline measurement kinetics, CTC quantification should also provide predictive information about therapeutic response, and aid clinicians in selecting the most appropriate treatment for each patient at each moment. In the SWOG S0421 study, changes in CTC levels from day 0 to day 21 were prognostic, where any increase in CTC counts as a continuous variable from day 0 to day 21 was associated with reduced OS. However, decreases in CTC count showed only a trend towards improved OS [81]. The authors suggested that an early decrease at day 21 after treatment onset could not be maintained over time and therefore renders it difficult to determine the actual prognostic implications of this CTC reduction, while an early increase after therapy initiation may reflect primary resistance and constitute a prognostic factor associated with poor outcome.

In a meta-analysis, Ma et al. confirmed the strong prognostic value of CTCs [82]. They demonstrated that CTC detection using immunohistochemistry was less accurate than using CellSearch or RT-PCR. Although only CellSearch has been approved by the FDA, RT-PCR performs as well as the CellSearch system in many cases. Nevertheless, any method can overlook EMT phenotype cells or CTC clusters. However, the complexity and heterogeneity of the CTC population requires the application of more versatile methods that allow deeper molecular characterisation than CellSearch.

At present, no robust data support the validity of CTC enumeration and patient outcome obtained using methods other than CellSearch, despite the promising results these technologies demonstrate in terms of sensitivity.

#### 4.2.4. Challenges to Actual Inclusion of CTC Analyses for Personalising Treatment of PCa

To introduce the use of CTC and the derived biomarkers in the day-to-day management of PCa, the necessity of proving the cost-efficiency of their analysis for reimbursement in many health systems should be borne in mind. It is important to note that although it received FDA approval in 2006, many insurance providers still consider CellSearch investigational and it has not been evaluated by agencies such as NICE or others. To improve the cost-effectiveness ratio of CTC analysis, the cost of CTC enumeration should be reduced and the molecular characterisation upgraded to increase the value of performing CTC enumeration in terms of quality-adjusted years gained.

In some clinical scenarios, the prognostic information provided by CTC analysis could change the clinical practice. For example, when clinicians have to determine if a patient will survive long enough to derive benefit from an immunotherapy that needs 3–6 months to be effective, or whether surgical palliation of spinal cord compression is guaranteed. In these cases, clinicians use prognostic estimators based on clinical and analytic parameters, but there is evidence that CTCs improve the predictive value, especially to decide phase I enrolment [83].

Even more important is the relationship between CTC increases and decreases within the course of the disease. CTC levels could aid oncologists in distinguishing early PSA and radiographic flare from actual progression, or when clinical deterioration without PSA changes is observed suggesting switch of the tumour to a neuroendocrine pathology. The higher correlation between CTC changes and survival in several phase III trials that investigated PSA levels supports this [9,10,81]. In the COU-AA-301 trial comparing abiraterone plus prednisone versus prednisone alone for patients with mCRPC, Scher and collaborators showed that combining CTC number and LDH level was a surrogate for survival at individual patient level [11]. However, PSA changes after treatment were not always prognostic in these studies and should not be used for selecting treatments.

Various groups have attempted AR analysis in CTCs with promising results (Table 2). Miyamoto et al. determined that the AR signalling status in CTCs from patients under androgen deprivation therapy was a possible indicator of therapy efficacy [56]. Reinforcing the value of AR analysis, recent studies have proposed the evaluation of AR modifications in CTCs, including the detection of AR-V7 and point mutations as an accessible and valuable tool for treatment selection [84]. AR-V7 detection in CTCs from men with mCRPC was not associated with primary resistance to taxane chemotherapy. In AR-V7-positive men, taxanes appear more efficacious than enzalutamide or abiraterone therapy, whereas taxanes and enzalutamide or abiraterone may have comparable efficacy in AR-V7-negative men [85]. Todenhöfer et al. used a sensitive whole-blood RT-PCR assay to assess AR-V7 expression in CTCs. In the validation cohort, patients with an AR-V7-positive result had lower PSA RR (0% vs. 42%, *p* = 0.27) and shorter median PSA-PFS (0.7 months vs. 4.0 months, *p* < 0.001) and median OS (5.5 months vs. 22.1 months, *p* < 0.001) [86]. However, some questions remain unanswered, and there are conflicting results in this field. For example, in the analysis of 21 AR-V7-positive patients with castration-resistant PCa who had received abiraterone or enzalutamide therapy, Bernemann et al. detected a subgroup of six AR-V7-positive patients who benefited from these agents. The authors concluded that AR-V7 status in CTCs cannot entirely predict nonresponse to next-generation androgen deprivation therapy [87].

Another study (NCT02485691) evaluated the correlation of a signature of resistance to AR-targeted agents with clinical outcome by analysing CTC phenotypes and expression and the localisation of proteins, including AR isoforms, in CTCs. A similar strategy would use CTC analysis to select the patients that would benefit from treatment and which patients are in progress and should switch to a new agent.

In this context, CTC analysis has also been used to guide treatment selection in some clinical trials. In breast cancer, human epidermal growth factor receptor 2 (HER2) expression on CTCs has been assessed using CellSearch, and other investigators are using CellSearch to monitor endocrine resistance in estrogen receptor (ER)-positive, HER2-negative metastatic disease (COMETI phase II trial, NCT01701050). In PCa, various studies are exploring the clinical applications of CTCs. In the multicentre, randomised, open-label phase II CABARESC study, the pharmacodynamic effects of budesonide on cabazitaxel were evaluated in patients with mCRPC. AR-V7 was detected in 16 of 29 patients (55%) with ≥10 CTCs per 7.5 mL blood and was more frequently found in abiraterone-pretreated patients (5 of 5 (100%) treated vs. 7 of 20 (35%) untreated; *p* = 0.009). There were no differences in CTC and PSA RRs. Importantly, the presence of AR-V7 in CTCs was not associated with progression-free survival (HR: 0.8; 95% confidence interval [CI], 0.4–1.8) or OS (HR 1.6; 95% CI, 0.6–4.4), suggesting that response to cabazitaxel is independent of CTC AR-V7 status [84].

One interesting clinical application of the differences in tumour biology between high- and low-CTC groups is the evaluation of new treatments, especially for strategies that may be associated with more toxicity only in the high-CTC subgroup. With this approach, potential toxicities could be minimised, the number of patients who derive benefit would be reduced, and patients can be treated according to their underlying biological profile. Examples of studies on this high-CTC subpopulation are the NCT01499043 study, where only patients with >10 CTCs per 7.5 mL blood detected by baseline CellSearch analysis were included in the study and treated with the multi-targeted tyrosine kinase inhibitor PLX3397. A 10-CTC per 7.5 mL blood threshold was also used in the NCT00887640 trial that studied the effect of temsirolimus in CPRC.

CTCs have also found a place as surrogate biomarkers in preclinical testing. CTC enumeration has the appeal of being an early and straightforward test for activity and also the potential of providing pharmacodynamic information [7]. A pharmacodynamic biomarker indicates “that there is a direct pharmacological effect of a drug”, but it does not necessarily yield prognostic or predictive information [89]. The value of this test is that it allows measurement of the treatment effect on its targets; if treatment foes not affect a reliable pharmacodynamic test, the treatment will be ineffective. This ability to predict non-efficacy early in the clinical development of a drug candidate, and the possibility of repeating CTCs enumeration at different time points within the development of the disease without the problems associated with repeated biopsies, has the potential for reducing costs and timelines and improving the rate of success of the next generation of clinical trials [90,91].

## 5. Conclusions

**Figure 1:**
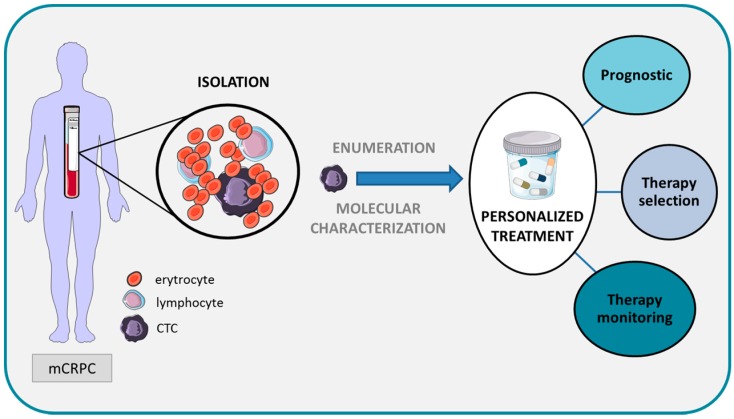
Schematic representing the value of CTCs for achieving precision oncology in patients with prostate cancer.

CTC analysis is one of the most challenging fields in translational oncology research. CTCs are considered tumour liquid biopsies that are of great value in providing information on PCa detection, prognosis, prediction and therapy response (Figure 1). Several studies have demonstrated surrogacy between CTC count and outcome in both localised and metastatic PCa. However, to determine the true validation of CTC detection in clinical practice, efforts to develop coordinated multicentre studies that include larger cohorts with longer observation periods, and more standardised and sensitive detection methods, need to be attempted. Currently, the CellSearch system is the standard method for CTC detection for clinical applications. Many new technologies are being developed for CTC detection and molecular profiling but their clinical utility should be demonstrated and compared with FDA-approved technology. CTC molecular characterisation is of great relevance for developing novel anti-cancer compounds, increasing in our knowledge of the mechanisms involved in metastatic development and also for predicting therapy activity, providing great promise for the future management of patients with PCa.

In conclusion, although the clinical significance of CTC detection has been demonstrated in many clinical studies so far, much work remains to be done to accredit the analytical methodologies used for CTC isolation, detection and molecular characterisation before their implementation in the routine clinic in order to provide personalised medicine for patients with PCa. It is important to keep in mind that CTC analysis has the potential to become one of the most promising oncology tests.

## Figures and Tables

**Table 1:** Systems used for isolating CTCs in patients with PCa.

System	Isolation Strategy	Identification	Referene
CellSearch	Immunocapture (EpCAM)	IF for CK and CD45, and DAPI	[24]
MagSweeper	Immunocapture (EpCAM, CD45)	PCR for *PSA*, *KLK3*, *TMPRSS2*, *CD45*	[22]
EPISPOT assay	Non-EpCAM-based immunocapture of CD45- and CXCR4-positive cells	Secretion of proteins: CK19, MUC1, PSA	[25]
ISET	Cell size	ICC for CK	[26]
Metacell	Cell size	ICC or IF for CK	[27]
ApoStream	Dielectrophoretic field	ICC for EpCAM and CK	[28]
CTC membrane microfilter	Cell size	IF for CK	[29]
DEPArray	Microfluidic and dielectrophoretic field	Image-based selection	[30]
CellCollector	In vivo immunoisolation (EpCAM)	IF for CK, EpCAM, CD45	[31]
Ficoll-Paque	Cell density	ICC for CK, PSA PCR	[32]
Vita-Assay	Marker-independent functional collagen adhesion matrix	ICC or flow cytometry (EpCAM, CK, CD44, CD34, CD45, vimentin)	[33]
RosetteSep	Immunodepletion of CD45-positive cells	IF for CK, EpCAM, CD45	[34]
AdnaTest	Immunocapture (EpCAM or EMT markers)	qRT-PCR	[23]
NanoVelcro CTC Chip	Microfluidics and immunocapture	IF for CK, EpCAM, CD45	[35]
GEDI microfluidic device	Microfluidics/immunocapture (PSMA)	ICC for CD45, PSMA, EpCAM	[36]
CTC-iChip	Microfluidic and immunocapture	Immunofluorescence, cytopathology, FISH	[37]

**Table 2:** Clinical response of patients with PCa according AR-V7 status.

Ref.	Treatment	AR-V7 Prevalence	PSA Response in AR-V7^+^ vs. AR-V7^−^ Patients	AR-V7 Assay
Antonarakis et al. [53]	Abiraterone, enzalutamide	19% 39%	0% vs. 68% (*p* < 0.01) 0% vs. 53% (*p* < 0.01)	CTC-derived mRNA
Steinestel et al. [88]	Abiraterone or enzalutamide	64%	7% vs. 63% (*p* < 0.01)	CTC-derived mRNA
Todenhöfer et al. [86]	Abiraterone	11%	0% vs. 42% (*p* < 0.01)	Whole-blood mRNA
Antonarakis et al. [85]	Docetaxel or cabazitaxel	46%	41% vs. 65% (*p* < 0.01)	CTC-derived mRNA
Onstenk et al. [84]	Cabazitaxel	55%	8% vs. 22% (*p* < 0.01)	CTC-derived mRNA
Scher et al. [54]	Abiraterone, enzalutamide or taxanes	18%	0% vs. 64% 33% vs. 44%	CTC-derived mRNA
